# Photoinduced dynamics of a cyanine dye: parallel pathways of non-radiative deactivation involving multiple excited-state twisted transients

**DOI:** 10.1039/c4sc02881c

**Published:** 2015-02-11

**Authors:** Srigokul Upadhyayula, Vicente Nuñez, Eli M. Espinoza, Jillian M. Larsen, Duoduo Bao, Dewen Shi, Jenny T. Mac, Bahman Anvari, Valentine I. Vullev

**Affiliations:** a Department of Bioengineering , University of California , Riverside , CA 92521 , USA . Email: vullev@ucr.edu; b Department of Biochemistry , University of California , Riverside , CA 92521 , USA; c Department of Chemistry , University of California , Riverside , CA 92521 , USA; d Materials Science and Engineering Program , University of California , Riverside , CA 92521 , USA

## Abstract

Cyanine dyes are broadly used for fluorescence imaging and other photonic applications. 3,3′-Diethylthiacyanine (THIA) is a cyanine dye composed of two identical aromatic heterocyclic moieties linked with a single methine, –CH=. The torsional degrees of freedom around the methine bonds provide routes for non-radiative decay, responsible for the inherently low fluorescence quantum yields. Using transient absorption spectroscopy, we determined that upon photoexcitation, the excited state relaxes along two parallel pathways producing three excited-state transients that undergo internal conversion to the ground state. The media viscosity impedes the molecular modes of ring rotation and preferentially affects one of the pathways of non-radiative decay, exerting a dominant effect on the emission properties of THIA. Concurrently, the polarity affects the energy of the transients involved in the decay pathways and further modulates the kinetics of non-radiative deactivation.

## Abstract 2

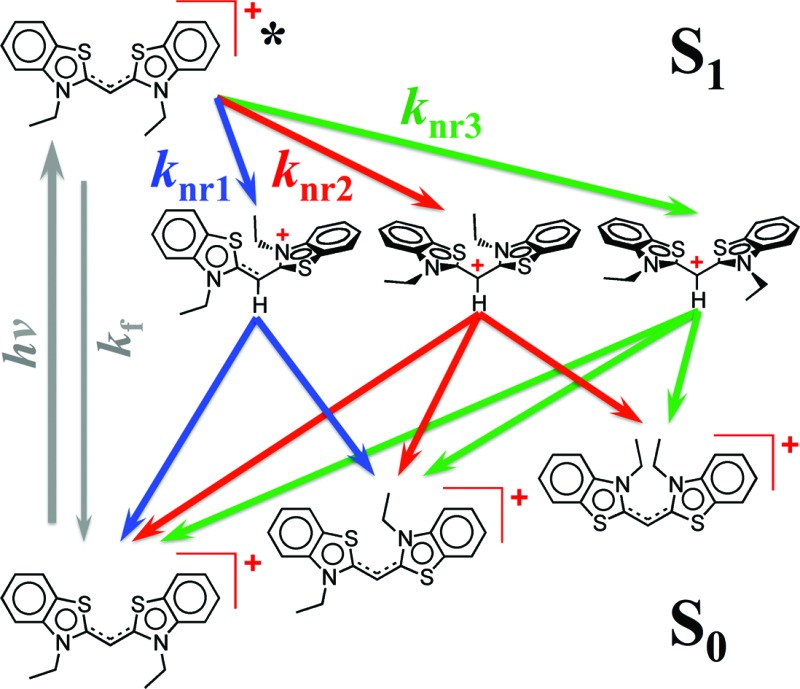
A photoexcited cyanine dye deactivates *via* multiple non-radiative pathways, only one of which is principally responsible for quenching its fluorescence.

## A. Introduction

**Scheme 1:**
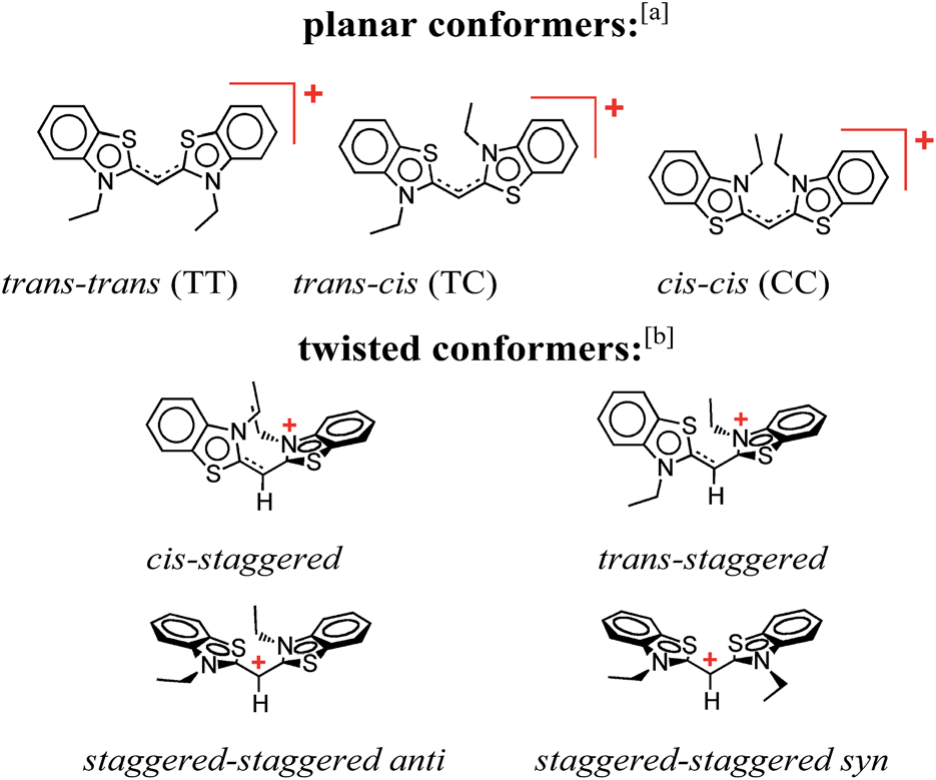
Conformers of 3,3′-diethylthiacyanine (THIA). ^a^ Although *cis*/*trans* nomenclature has been adopted for the planar conformers of THIA and other cyanine dyes, it is not quite accurate. Conversely, the *E*/*Z* nomenclature should be used to account for the four different substituents of the two partially π-conjugated bonds of the methine linker, *i.e.*, *trans–trans* should be *Z–Z* (S has higher priority than N, and C has higher priority than H), *trans–cis* – *Z*–*E*, and *cis*–*cis* – *E*–*E*. ^b^ For a bond between sp^2^ hybridized carbons, *staggered* implies about 90° dihedral angle, unlike 60°, 180° and 300° for a bond between sp^3^ hybridized carbons.

This publication describes a study revealing the complex photophysics of 3,3′-diethylthiacyanine (THIA). THIA is one of the simplest cyanine dyes (Scheme 1), and its inherent properties as an oligomethine chromophore have defined its use in biological imaging, nanomaterials, and photonics.^1^–^6^ Despite the relative structural simplicity of this cyanine dye, its excited- and ground-state dynamics manifest complexity that can strongly affect its utility for the different applications.

Oligo- and polymethine structures contain an odd number of –CH= groups providing a means for extending π-conjugation over alternating single and double bonds. Oligomethine is the main structural motif in a range of chromophores and redox active cofactors, such as carotenoids, cyanine dyes, and retinal. Extending the π-conjugation tunes the optical properties of the oligomethines, and pushes their absorption and emission to the near infrared (NIR) region of the spectrum. As an example, indocyanine green (ICG), a cyanine dye with an elongated polymethine chain, is the only NIR dye approved by the U. S. Food and Drug Administration (FDA) for specific medical imaging applications.^7^–^9^

Carotenoids are vital components of the photosynthetic reaction centers, where they prevent photodamage by various mechanisms including participation in regulatory pathways, and serve as auxiliary chromospheres for utilizing solar energy in the mid-range of the visible spectrum.^10^–^14^ Their elongated molecular structure and high susceptibility to oxidation have made carotenoids preferred components for artificial light harvesting systems.^15^–^19^

Cyanine dyes, comprising an oligomethine that links two aromatic moieties, are broadly used photoprobes for biochemical and biomedical applications.^20^–^23^ Controlling the extent of π-conjugation allows for tuning the photophysical properties of these oligomethine structures throughout the visible and NIR spectral regions. The charge delocalized over the π-conjugated systems, and the inherently hydrophobic character of the hydrocarbon structures provide the amphipathicity of cyanine dyes, which governs their high propensity for aggregation and staining. While the extended π-conjugation carries a positive charge, the addition of side chains with acidic groups, such as sulfonates, results in zwitterionic or negatively charged cyanine dyes.^24^–^28^ The propensity for aggregation and the high affinity of the positively charged cyanines for negatively charged macromolecular templates is key for their utility as photoprobes for structural analysis.^29^–^32^

The inherent flexibility of the oligomethines, however, results in considerable complexity in the photophysics of cyanine dyes and other conjugates with a similar structure. Indeed, extending the length of the methine linkers ensures a decrease in the HOMO–LUMO energy gap and red shifts in the absorption and emission spectra. Attaining such red spectral shifts, however, requires a planar or close to planar molecular conformations. While the π-conjugation stabilizes planar conformation, entropically driven dynamics and steric hindrance favour structures with twists in the oligomethine chains. Such twisted structures are especially pronounced in the electronically excited states where the π-bonding character of the methine linkers is diminished. The twisting dynamics and breaking of the π-conjugation provides pathways for non-radiative deactivation of excited states causing a drastic decrease in the emission quantum yields of cyanine dyes. For example, while NIR absorption and fluorescence of ICG are immensely favourable for biological imaging,^33^–^36^ its fluorescence quantum yield of a few percent presents a principal challenge in the development of medical applications. Conversely, understanding the effects of the torsional modes of deactivation on the fluorescence quantum yields of cyanine dyes can prove beneficial for biological applications. For example, twisting modes of deactivation result in negligible fluorescence quantum yields of many chromophores when in non-viscous aqueous media.^37^–^40^ Binding of such chromophores to rigid macromolecular matrixes can readily restrict their twisting degrees of freedom, thus, resulting in enhancement of the fluorescence quantum yield up to several orders of magnitude. Under such circumstances, the emission signal comes predominantly from the stained macromolecular components of cells or tissue structures and not from the free dye molecules in the surrounding environment, thereby facilitating high-contrast imaging of biological samples.^41^–^46^

THIA is one of the simplest cyanine dyes with a single methine linking two identical benzothiazole moieties (Scheme 1). Based on the geometry of the two bonds of the linker, THIA has three planar isomers (Scheme 1): *trans*–*trans* (TT), *trans*–*cis* (TC) and *cis*–*cis* (CC). As expected from steric-hindrance considerations, TT is the most stable and CC is the least stable of the three planar structures.^47^–^50^ The torsional transitions between the three planar isomers involve twisted structures (Scheme 1), which in the ground electronic state are at the potential-barrier maxima, *i.e.*, they are transition states. In the lowest electronically singlet excited state, however, the twisted conformers are relatively stable structures at the bottom of potential-energy wells.^51^ Indeed, when we refer to planar conformers, we also include structures that are close to planar. For example, the two aromatic moieties of the *trans*–*trans* excited state of THIA that is at a local minimum, ^1^D*TT, are slightly out of plane due to a decreased bonding character of the π-conjugation favouring the planar structures.^50^,^51^ Keeping this feature in mind, ^1^D*TT is still referred to as a planar conformer.

**Scheme 2:**
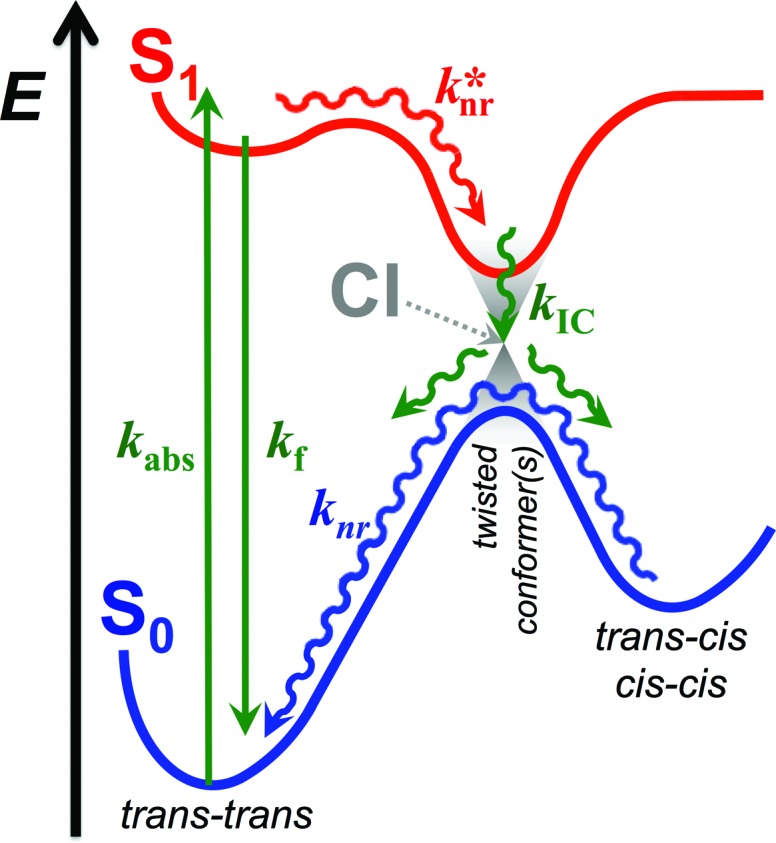
Generalized photoinduced dynamics of THIA and other cyanine dyes. Green arrows represent transitions between the ground, S_0_, and the excited state, S_1_, including radiative transitions: absorption, *k*_abs_, and fluorescence, *k*_f_; and non-radiative transition: internal conversion (IC), *k*_IC_. Red arrow represents non-radiative transition within the excited state, k*nr, encompassing rotation around the partially π-conjugated linker bonds, along with vibrational relaxation. Blue arrow represents non-radiative pathways within the ground state, *k*_nr_, encompassing the transitions of the two *cis* rotamers to the *trans*–*trans* conformer. CI = conical intersection.

The relative stability of the electronically excited twisted conformers and their lack of stability when in ground state results in a region of vibronic overlap between the S_1_ and S_0_ states, referred to as a conical intersection (CI), which provides a main pathway for efficient internal conversion (IC) leading to a non-radiative decay. Therefore, upon photoexcitation, THIA relaxes to a local minimum, ^1^D*TT, from where it can undergo radiative decay, *k*_f_; or *via* twisting modes it migrates to the CI, k*nr (Scheme 2). Upon IC, THIA relaxes to one of three ground-state planar isomers (Schemes 1 and 2). Ground-state thermally driven rotations around the methine-linker bonds lead to the most stable *trans*–*trans* conformer (Scheme 2).

This generally accepted mechanistic notion (Scheme 2) about chromophores with π-conjugated linker, however, does *not* feasibly represent the photophysics of THIA. As such, theoretical and experimental studies have shown that the relaxation of such a cyanine dye from its *trans*–*trans* S_1_ state, leads to the formation of all three planar isomers in the S_0_ state.^47^,^51^ The photoexcitation of THIA from its ground state leads only to the *trans*–*trans* excited-state isomer.^47^–^51^ It is not plausible to provide a single pathway of ^1^D*TT*via* one unique twisted structure at the CI that would lead to all three ground-state planar conformers. An increasing number of studies show multiple pathways of relaxation of cyanines along their excited-state potential-energy planes.^47^,^52^–^55^ The limit of a single S_1_ → S_0_ conical intersection, to which the multiple relaxation pathways are postulated to lead, however, sets challenging mechanistic constrains. Alternative rationales involving, for instance, an additional “hot” ground state remain somewhat physically unfeasible.^47^ Multiple conical intersections or an “extended seam”^52^ can account for the complex kinetics of S_1_ → S_0_ deactivation of THIA and other cyanine dyes. Showing a parallel involvement of multiple twisted conformers (Scheme 1) in the S_1_ → S_0_ decay would, indeed, provide unequivocal experimental evidence for this alternative mechanism.

Herein, we report transient-absorption studies of THIA for alcohol solutions of varying polarity and viscosity. Transients, absorbing in the mid-visible and UV spectral regions (around 530 nm and 360 nm, respectively), manifest the behaviour of excited-state intermediates at the interface with the ground-state potential-energy plane. Indeed, identifying the transient spectra of these twisted conformers is key for gaining mechanistic insights about the non-radiative pathways of deactivation of the photoexcited dye. Dependence of the transient-absorption kinetics of THIA on the media viscosity and polarity allows for identifying two parallel pathways of excited-state non-radiative deactivation that lead to three points of internal conversion to the ground state. Relaxation after IC produces the three planar conformers. An increase in media viscosity tends to suppress the IC pathway leading directly to the *trans*–*trans* conformer. We identified this direct transition to be principally responsible for quenching the THIA emission.

## B. Results

### B.1. Structural considerations

**Scheme 3:**
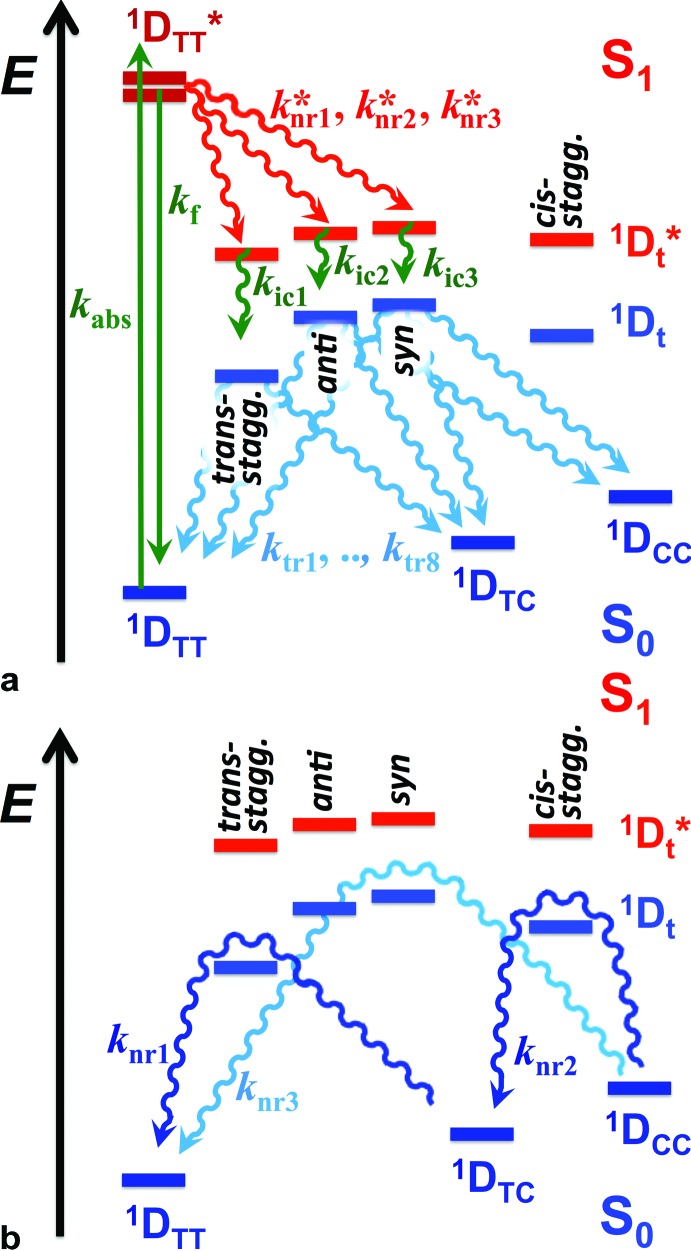
Multiple paths of relaxation of THIA. (a) Deactivation of the excited state, involving non-radiative pathways, represented with k*nr, leading to the three ground-state planar conformers (Scheme 1) *via* internal conversion (*k*_ic_) and torsional relaxation (*k*_tr_). (b) Ground-state thermal relaxation of the two *cis* rotamers, ^1^D_TC_ and ^1^D_CC_, to the most-stable *trans–trans* conformer, ^1^D_TT_ (Scheme 1). Experimentally, *k*_nr*i*_ are determined from the decays of the ^1^D*TT and the rise of the ^1^D*t transients, which include the decays of the 720 nm transient, *k*_TT_, the stimulated emission, *k*_SE_, and the transient “hidden” in the bleach band, *k*(d)B; and the rise of the 530 nm transient, *k*(r)t. *k*_ic*i*_ are determined from the decays of the ^1^D*t transients observed at 530 and 360 nm, *i.e.*, from *k*(d)t and *k*_360 nm_. *k*_tr*i*_ were related to the rise of the 450 nm absorption band of the two ground-state *cis* isomers, *k*(r)C, and the fast rise (depletion) of the bleach band, *k*(r)B1. *k*_nr*i*_ were determined from the decay of the *cis*-isomer band, *k*(d)C, and the slow rise components of the bleach band *k*(r)B.

Despite the numerous possible twisted conformers of the cyanine dye, not all of them can be feasibly involved in the excited- and ground-state relaxation. Photoexcitation of THIA generates S_1_ with *trans*–*trans* character. Rotation around one of the linker bonds produces only one of the excited-state twisted conformers, ^1^D*t, *i.e.*, the *trans-staggered* one (Scheme 3a). For obtaining a *cis-staggered* structure, an additional 180° rotation of the other ring is required. Such an additional rotation ought to proceed through the *syn* or the *anti staggered–staggered* rotamers (Scheme 1). Following internal conversion to S_0_, the *trans-staggered* twisted structure can produce the *trans*–*trans*, ^1^D_TT_, and *trans*–*cis*, ^1^D_TC_, ground states *via* additional 90° ring rotation. The formation of the *cis*–*cis* planar conformer, ^1^D_CC_, requires rotation around both bonds of the linker.

Concerted 90° conrotatory and disrotatory turns around both linker bonds lead to the *staggered*–*staggered anti* and *syn* twisted conformers, respectively (Scheme 1). Disrotatory relaxation of the *syn* twisted rotamer yields the *trans*–*trans* and *cis*–*cis* planar conformers, depending on the direction of rotation; and conrotatory relaxation of the *staggered*–*staggered syn* structure leads only to ^1^D_TC_. Conrotatory relaxation of the *anti* rotamer provides the pathway to the planar ^1^D_TT_ and ^1^D_CC_, while the disrotatory relaxation of the *anti* leads to ^1^D_TC_. Therefore, relaxation *via* rotations around both linker bonds and transitions through the *staggered–staggered* structures (Scheme 3a) provides the only possible S_1_ → S_0_ pathways to all three planar conformers, ^1^D_TT_, ^1^D_TC_ and ^1^D_CC_.

Once the ground-state *cis* structures form, thermally relaxation leads to the most stable *trans*–*trans* conformer (Schemes 2 and 3b). Rotation around only one of the linker bonds can readily account for the transition from ^1^D_TC_ to ^1^D_TT_*via* the ground state of the *trans-staggered* twisted conformer (Scheme 3b). The ground-state conversion of *cis–cis* to the *trans–trans* isomer, however, can occur *via* either (1) a stepwise pathway, ^1^D_CC_ → ^1^D_TC_ → ^1^D_TT_, involving rotations of only one of the rings at a time, proceeding through the *cis-staggered*^1^D_t_ for the first step and *via* the *trans-staggered* for the second (Scheme 3b); or (2) a concerted transformation, ^1^D_CC_ → ^1^D_TT_, involving simultaneous rotation of both rings, proceeding through the *staggered–staggered syn*^1^D_t_ (Scheme 3b).

Overall, the consideration of the plausible torsional modes of THIA, results in a complex scheme of deactivation of the photoexcited dye (Scheme 3). Three alternative pathways of non-radiative deactivation of ^1^D*TT (k*nr) lead to three different twisted excited states, ^1^D*t, that undergo internal conversion, *k*_ic_, to produce the corresponding twisted ground states, ^1^D_t_ (Scheme 3a). Through eight possible pathways of torsional relaxation, *k*_tr_, the three ^1^D_t_ transform into the three planar ground-state conformers, ^1^D_TT_, ^1^D_TC_ and ^1^D_CC_. This complex kinetic scheme can hardly fit into the accepted notion for deactivation of photoexcited cyanine dyes (Scheme 2).

Using Scheme 3 as a set of working hypotheses, we present a detailed investigation of the transient-absorption kinetics of THIA. Quantitative analysis permits relating the experimentally determined rates with the hypothetical rate constants as shown on Scheme 3. Following the introduction of the photophysics of THIA (Section B.2) and the assignments of its transient-absorption features (Section B.3), we present kinetic analysis of the deactivation of the photoexcited dye in ethanol media (Sections B.4). The effects of solvent viscosity and polarity reveal key trends in the relaxation dynamics of THIA (Section B.5) that allow us to propose a kinetic scheme similar to Scheme 3 but based on experimentally determined rate constants (Section C). Furthermore, the solvent effects pointed toward the non-radiative deactivation pathways that most distinctly compete with the radiative decay, *k*_f_, and are responsible for the media dependence of the emission quantum yield of THIA (Section C).

### B.2. Photophysical considerations

**Fig. 1:**
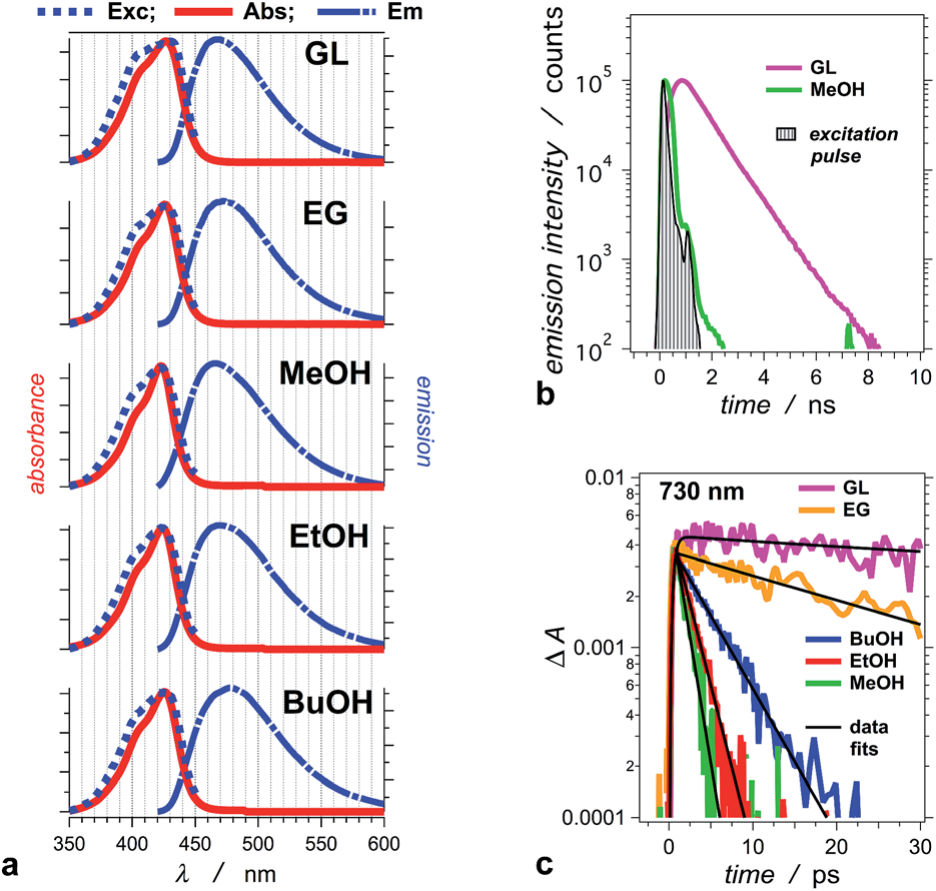
Photophysical properties of THIA in: glycerol (GL), ethylene glycol (EG), methanol (MeOH), ethanol (EtOH), and 1-butanol (BuOH). (a) Normalized steady-state spectra: absorption (Abs), emission (*E*_m_, *λ*_ex_ = 400 nm), and excitation (*E*_xc_, *λ*_em_ = 470 nm). (b) Emission decays for glycerol and methanol recorded using TCSPC (*λ*_ex_ = 406 nm, *λ*_em_ = 475 nm, half-height pulse width, PW = 195 ps). (c) Transient-absorption decays of THIA in the five alcohols, recorded at 730 nm. This transient corresponds to the excited-state conformer that undergoes radiative deactivation (*λ*_ex_ = 385 nm; PW_800 nm_ = 50 fs, 2 μJ per pulse).

THIA absorbs at the blue edge of the visible spectrum (*i.e.*, *λ*_abs_ < 450 nm), and with a Stokes shift of 40 to 50 nm, its fluorescence spreads between about 450 and 550 nm (Fig. 1a). We selected five alcohols, with different polarities and viscosities, as a media to investigate the photophysics of THIA. While glycerol is the most polar and the most viscous of the five solvents, 1-butanol is the least polar and methanol is the least viscous (Table 1). Solvation by polar media stabilizes the twisted structures with partial charge localization (Scheme 1). Therefore, polarity dependence of the photophysics of THIA would suggest rate-limiting steps governed by the energy of the twisted states. Conversely, the media viscosity impedes the molecular motions, and especially the modes of rotation of the aromatic rings around the methine linker bonds. Thus, if the transitions between the various rotamers have a rate-limiting effect on its photoinduced dynamics, photophysics of THIA should pronouncedly depend on the solvent viscosity.

**Table 1:** Photophysical properties of THIA for alcohol media with different polarity and viscosity

Solvent^*a*^	*ε* ^*b*^	*η* ^*c*^/cP	*Φ*_f_ ^*d*^ × 10^2^	*τ*_TT_ ^*e*^/ps	*k*_f_ ^*f*^ × 10^–8^/s^–1^	*λ*_abs_ ^*g*^/nm	*λ*_em_ ^*g*^/nm	Δ*λ*^*h*^/nm
GL	43	930	16	659 ± 52;^*i*^ 913 ± 26^*j*^	2.4;^*i*^ 1.8^*j*^	427	468	41
EG	41	17	0.63	30.2 ± 1.6	2.1	426	473	46
MeOH	33	0.55	0.045	1.46 ± 0.16	3.1	423	470	47
EtOH	24	1.2	0.077	2.25 ± 0.08	3.4	424	470	46
BuOH	18	2.5	0.16	5.05 ± 0.53	3.2	425	478	53

^*a*^GL = glycerol; EG = ethylene glycol; MeOH = methanol; EtOH = ethanol; BuOH = 1-butanol. Except GL and EG solutions, all samples were purged with argon.

^*b*^Relative static dielectric constant of the neat solvent.

^*c*^Dynamic viscosity of the neat solvent.

^*d*^Emission quantum yield (*λ*_ex_ = 385 nm).

^*e*^Decay time constant of the *trans*–*trans* excited state from the transient absorption decay monitored at 730 nm (Fig. 1c).

^*f*^Apparent radiative-decay rate constant, *k*_f_ = *Φ*_f_/*τ*_TT_.

^*g*^Wavelengths of the absorption and emission spectral maxima.

^*h*^Stokes' shift: Δ*λ* = *λ*_em_ – *λ*_abs_.

^*i*^From transient absorption decay (Fig. 1c).

^*j*^From TCSPC (Fig. 1b).

Hydrogen bonding properties of solvents can affect the non-radiative modes of deactivation of excited electronic states and alter the photophysics of photoprobes.^56^–^60^ Mediated by high-energy O–H vibrations, improved coupling between the S_1_ and S_0_ states provides efficient pathways for internal conversion. It is the most frequently encountered effect of fluorescence quenching attributed to protic media. Alternatively, hydrogen bonding to carbonyls may alter the energy of n → π* states, leading to impeded intersystem crossing and enhanced fluorescence quantum yields.^61^–^63^ The solvent propensity for hydrogen bonding can also affect the emission of cyanine dyes. The photophysics of cyanine dyes that contain hydrogen-bond accepting groups, such as sulfonates, exhibit a noticeable D_2_O/H_2_O isotope effect.^64^ As determined from fluorescence quantum yields and lifetimes, this isotope effect becomes substantial (*i.e.*, >1.1) only when the *oligo*-methine linker comprises more than three carbons. Because THIA has only a single methine carbon and does not contain hydrogen-bonding groups, we did not expect to detect effects due to the proticity of the media. Using acetonitrile, an aprotic solvent that is more viscous and slightly more polar than methanol, we examined if hydrogen-bonding propensity of the media affects the photophysics of THIA.

**Fig. 2:**
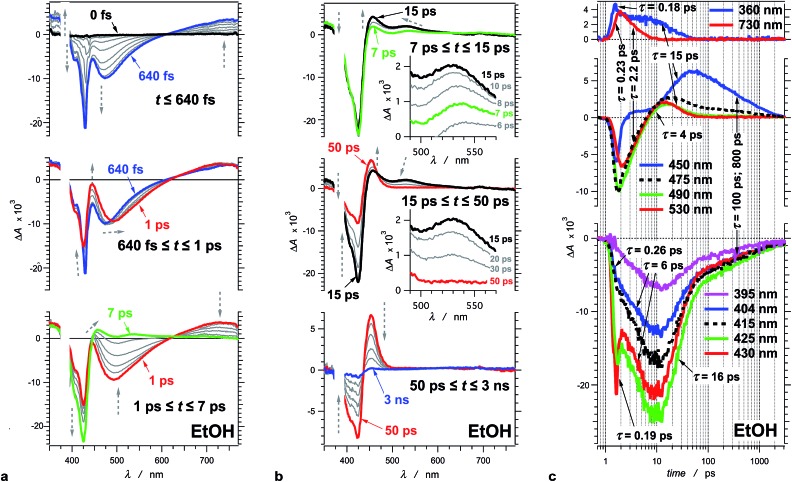
Transient absorption of THIA (10 μM in ethanol). (a) Chirp-corrected transient-absorption spectra in the subpicosecond and picosecond time domains. (b) Transient-absorption spectra in the picosecond and nanosecond time domains (insets). Expanded spectral region around 530 nm, showing the band that was ascribed to the twisted conformers of the singlet excited state. The grey dashed arrows indicate the changes in Δ*A* (vertical direction) and the shifts of the spectral maxima and minima (horizontal direction). (c) Transient-absorption kinetics recorded at different wavelengths. The logarithmic abscissa allows for presenting the various time domains on the same graph. The pump time, *i.e.*, *t*_0_, was set at 1 ps. (*λ*_ex_ = 385 nm; PW_800 nm_ = 50 fs, 2 μJ per pulse).

For the series of alcohols with different polarities, THIA exhibits negligible solvatochromism. Conversely, viscosity of the solvents strongly affects the fluorescence quantum yield, *Φ*_f_, and the lifetime of the dye (Table 1).^2^,^65^ For all solvents, but glycerol, the emission lifetime of THIA is too short (*i.e.*, <100 ps) to be reliably measured using time-correlated single photon counting (TCSPC). A transient, absorbing between about 650 and 800 nm (Fig. 2), is ascribed to the *trans–trans* excited-state conformer of THIA that is responsible for its radiative deactivation.^51^ Therefore, we use the transient-absorption decays, recorded at 730 nm, for estimating the emission lifetime of THIA: *i.e.*, the lifetime of its *trans–trans* excited state, *τ*_TT_ (Fig. 1c). While TCSPC-recorded emission for glycerol manifests a single exponential decay (Fig. 1b), we detect biexponential kinetics for the deactivation of the 730 nm transient. Indeed, short picosecond components with small contributions to the decay kinetics may remain undetectable by TCSPC. For glycerol, the 730 nm biexponential decay kinetics encompasses *τ*_1_ = 775 ± 86 ps (*α*_1_ = 0.81), *τ*_2_ = 63.9 ± 21.5 ps (*α*_2_ = 0.19). For a non-viscous solvent, acetonitrile, the fast component of the 730 nm biexponential decay has a major contribution to the kinetics: *τ*_1_ = 1.28 ± 0.12 ps (*α*_1_ = 0.96), *τ*_2_ = 33.3 ± 4.7 ps (*α*_2_ = 0.04). Because of the relatively low signal-to-noise ratios for most of the 730 nm decays, however, we cannot always observe a statistically significant improvement for the use of bi- *vs.* monoexponential data fits. Therefore, we report the simplest, *i.e.*, monoexponential, results for *τ*_TT_.

Similar to *Φ*_f_, *τ*_TT_ of THIA shows dependence on the media viscosity, rather than on the polarity, resulting in similar emission rate constants, *k*_f_, for the different solvents (Table 1). Also, while the time constants of radiative decay, *k*_f_^–1^, are in the order of nanoseconds, the lifetimes of THIA are in the picosecond time domain (Table 1). Thus, non-radiative pathways, most likely involving transitions between different rotamers, govern the observed photophysics of THIA.

### B.3. Assignments of the transient-absorption spectral features

To illustrate the photoinduced excited-, S_1_, and ground-state, S_0_, dynamics of this cyanine dye, we focus on the evolution of the transient absorption of THIA dissolved in ethanol. Excitation at the blue edge of the S_1_ ← S_0_ absorption band leads to a subpicosecond growth of four features in the UV/visible spectral region (Fig. 2a). (1) A UV absorption band, at about 360 nm, is ascribed to singlet-excited-state transients.^66^ (2) A sharp narrow bleach band, which peaks at about 430 nm, corresponds to the depletion of the absorption of the ground-state *trans*–*trans* conformer, ^1^D_TT_. (3) A broad band with negative Δ*A* around 475 nm results from the stimulated emission. (4) A broad band around 730 nm corresponds to a singlet-excited-state *trans*–*trans* transient, ^1^D*TT.

In the timespan between 1 and 7 ps after the excitation, the transient-absorption band at 730 nm, along with the stimulated emission signal, decays to the baseline exhibiting isosbestic point at 627 nm (Fig. 2a). Concurrently, the magnitude of the bleach band, corresponding to the depleted *trans–trans* ground state, increases with about 50%, while the transient absorption at 360 nm remained practically unaltered (Fig. 2a).

At about 7 ps after the excitation, the inception of transient-absorption bands at 450 nm and 530 nm becomes apparent (Fig. 2b). The absorption band at 530 nm rises to reach its maximum value at about 12 ps, and subsequently decays along with the 360 nm band. In addition, the 530 nm band exhibits a blue shift during its rise and decay (Fig. 2b). Conversely, the 450 nm band rises pronouncedly during the decay of the transient absorption at 530 nm and 360 nm. The transient absorption at 450 nm reaches maximum at about 50 ps and then decays to zero leading to a recovery of the *trans–trans* ground-state absorption, as indicated by the decrease in |–Δ*A*| of the bleach band at 425 nm (Fig. 2b).

**Fig. 3:**
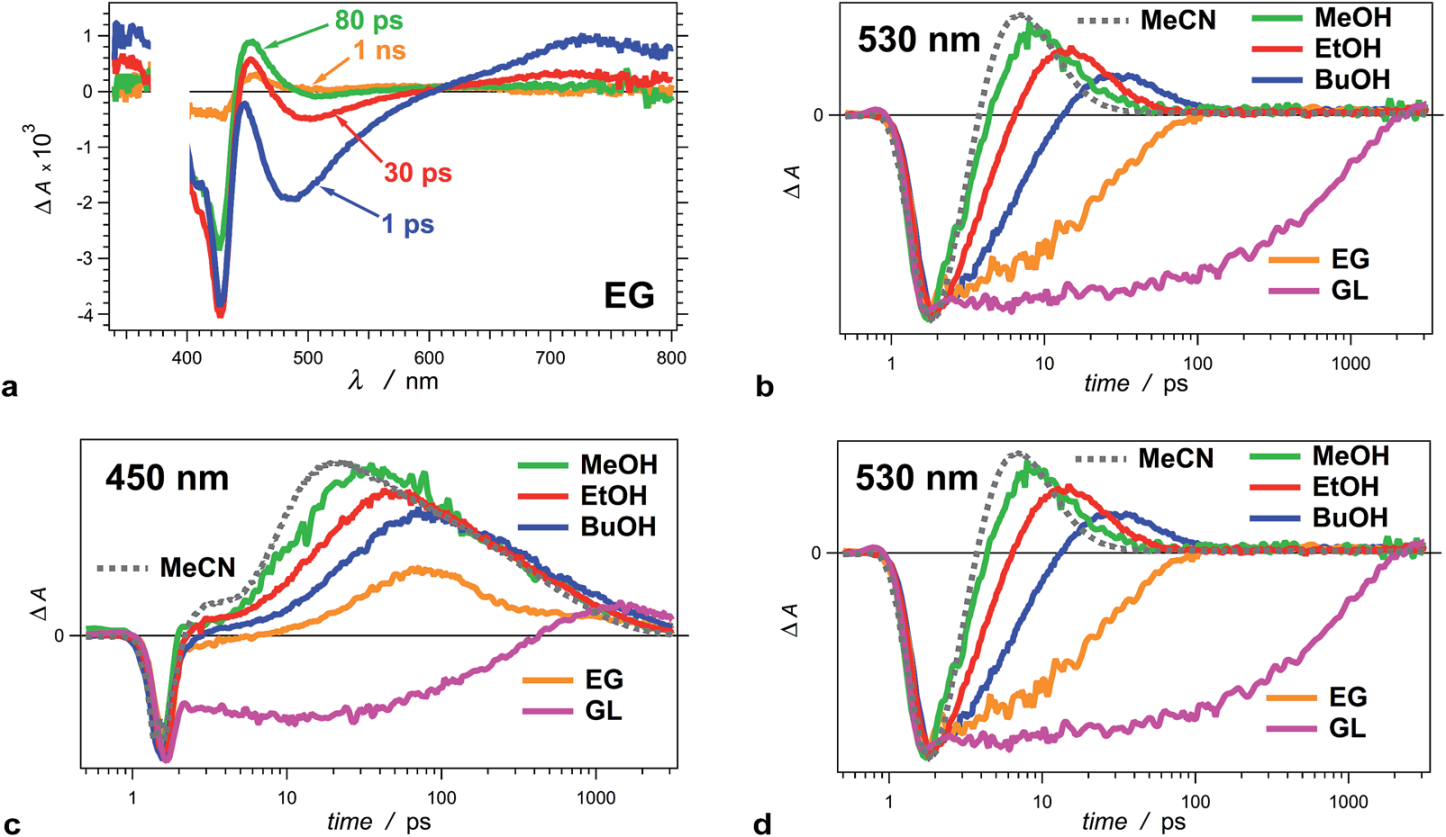
Solvent dependence of the transient-absorption kinetics of THIA (10 μM). (a) Chirp-corrected transient-absorption spectra for a relatively viscous solvent, ethylene glycol (EG). (b) Transient-absorption kinetics recorded at 530 nm (showing the rise and the decay of the twisted conformers) for alcohols with different polarity and viscosity (Table 1): methanol (MeOH), ethanol (EtOH), 1-butanol (BuOH), ethylene glycol (EG) and glycerol (GL), as well as for a non-viscous aprotic solvent, acetonitrile (MeCN). (c) Transient-absorption kinetics of THIA recorded at 450 nm (showing the formation and the decay of the ground-state *trans–cis* and *cis–cis* conformers) for different alcohols and for MeCN. (d) Transient-absorption kinetics of THIA recorded at peak (*i.e.*, minimum) of the bleach band (corresponding to the ground-state *trans–trans* conformers) for different alcohols and for MeCN. The logarithmic abscissa of the kinetic data allows for presenting the various time domains on the same graph. The pump time, *i.e.*, *t*_0_, was set at 1 ps. (*λ*_ex_ = 385 nm; PW_800 nm_ = 50 fs, 2 μJ per pulse).

This sequence of events was consistent with assigning the 450 nm transient to the ground-state *trans–cis*, ^1^D_TC_, and *cis–cis*, ^1^D_CC_, conformers of the dye (Scheme 1).^47^,^48^ We ascribe the 530 nm absorption to one or more conformers, ^1^D*t, involved in the non-radiative deactivation of the *trans–trans* excited state, ^1^D*TT, to the three ground-state rotamers, ^1^D_TC_, ^1^D_CC_, and ^1^D_TT_ (Scheme 3a). In accordance with theoretical simulations, ^1^D*t can be envisioned as twisted excited-state conformers (Scheme 1) at the intersections between the potential-energy planes of the S_1_ and S_0_ states.^50^ If the rates of formation of ^1^D*t, k*nr, are larger than the rates of internal conversion, *k*_ic_, leading to any of the ground-state conformers (Scheme 3a), a sufficient amount of this transient should accumulate allowing for monitoring its rise and decay. It is, indeed, the case for THIA in ethanol and other alcohols with relatively low viscosity (Fig. 3b).

Due to its concurrent decay with the stimulated emission, the broad 730 nm transient-absorption band is ascribed to a ^1^D*TT conformer, trapped in a local minimum, that undergoes radiative decay to the *trans–trans* ground state, ^1^D_TT_. The lack of significant change in Δ*A* at about 360 nm for the time between 1 and 7 ps indicates that this UV signal corresponds to the absorption (with similar extinction coefficients) of two or more transients that sequentially rise and decay. The decay of the 360 nm absorption (*τ*_360 nm_ = 15.4 ± 0.9 ps) becomes apparent at about 7 ps after the excitation, and is concurrent with the decay of the 530 nm band and with the rise of the 450 nm band. It suggests that at least one of the transients absorbing at 360 nm is also likely to be a twisted conformer, ^1^D*t, involved in the non-radiative deactivation to the ground states.

### B.4. Transient dynamics for ethanol media

#### B.4.1. Excited-state dynamics

The rate constant of the decay of the emissive ^1^D*TT is *k*_TT_ = 1/*τ*_TT_ = 4.5 × 10^11^ s^–1^ (Tables 1 and 2), which accounts for one, two or three of the non-radiative routes, as represented with k*nr, leading to the twisted conformers ^1^D*t (Scheme 3a). As determined from the growth of the 530 nm band, the rate constant of the formation of ^1^D*t is *k*(r)t = 1/*τ*(r)t = 2.0 × 10^11^ s^–1^ (Table 2), which should also relate to k*nr (Scheme 3a). Because the decay of ^1^D*TT is faster than the rise of ^1^D*t, *i.e.*, *k*_TT_ > *k*(r)t, another transient growth or bleach recovery (with *k* = *k*_TT_ – *k*(r)t = 2.5 × 10^11^ s^–1^, *i.e.*, *τ* = 4 ps) ought to accompany the deactivation of the emissive *trans–trans* conformer. The bleach recovery does not have any components with time constants close to 4 ps, indicating that a direct transition of ^1^D*TT to ^1^D_TT_ cannot account for this kinetic trend.

**Table 2:** Kinetic characteristics of the excited-state dynamics of THIA for different alcohol media, obtained from transient-absorption spectroscopy data^*a*^

Solvent^*b*^	*k*_TT_ ^*c*^ × 10^–10^/s^–1^	*τ*_360 nm_ ^*d*^/ps [*k*_360 nm_ × 10^–10^/s^–1^]	*τ*_SE_ ^*e*^/ps (*α*_*i*_) [*k*_SE_ × 10^–10^/s^–1^]	*τ* (r) t ^*f*^/ps [*k*(r)t × 10^–10^/s^–1^]	*τ* (d) t ^*g*^/ps [*k*(d)t × 10^–10^/s^–1^]
GL	1.6	1400 ± 300 [0.07]	77 ± 8 (0.20) [1.3]	—	—
	0.13		820 ± 30 (0.80) [0.12]		
EG	3.3	62 ± 9 [1.6]	25 ± 3 [4.0]	—	—
MeOH	67	8.9 ± 1.0 [11]	1.9 ± 0.4 [52]	2.5 ± 0.2 [40]	8.0 ± 0.7 [13]
EtOH	45	15 ± 2 [6.7]	2.2 ± 0.2 [45]	5.0 ± 0.2 [20]	13 ± 1 [7.7]
BuOH	20	31 ± 3 [3.2]	5.3 ± 1.1 [19]	8.8 ± 0.3 [11]	30 ± 2 [3.3]

^*a*^Time constants, *τ*, were extracted from exponential fits. For multiexponential data fits, 
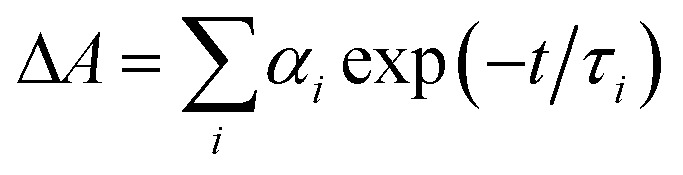
, the relative pre-exponential amplitudes of the same decay or the same rise, *α*_*i*_, are shown in the parentheses. The corresponding rate constants, *k* = 1/*τ*, are shown in the square brackets. The reported values were obtained from exponential data fits of the whole region (*i.e.*, from *t*_0_ = 1 ps to *t* = 3.2 ns). For EG and MeOH, when the data fits were limited to the temporal regions comparable with the expected time constants, the obtained values of *τ* were the same as the ones reported in ref. 51.

^*b*^GL = glycerol; EG = ethylene glycol; MeOH = methanol; EtOH = ethanol; BuOH = 1-butanol.

^*c*^From the time constants of the decays monitors at 730 nm (Table 1), *k*_TT_ = 1/*τ*_TT_.

^*d*^The slowest decay component of data fits of kinetic curves recorded at the near UV region, *i.e.*, at 360 nm.

^*e*^Decrease in the intensity of the stimulated emission, SE, from the fast rise component of data fits of kinetic curves recorded at 490 nm. The slow rise component at 490 nm was associated with the formation of the 530 nm transient, *τ*(r)t. For GL, the rise at 490 nm was biexponential (similar to the decay at 730 nm, Fig. 1b and Table 1) and the formation of a transient at 530 nm was not observed.

^*f*^Formation of the transient ascribed to the twisted conformer(s), D*t, from the slow rise component of data fits of kinetic curves recorded at 530 nm. The fast rise components had negligible contribution to the data fits, *i.e.*, small amplitudes, *α*_*i*_, and it was identical (within the uncertainty of the data fits) with *τ*_SE_.

^*g*^From the decays of the kinetic curves recorded at 530 nm.

An explanation for the difference between *k*_TT_ and *k*(r)t can involve an assumption that the absorption band at 530 nm belongs to more than one transient. That is, the shift of the 530 nm band (Fig. 2b) is due not only to conformational relaxation, but also to absorption overlap of multiple transients that rise and decay with slightly different rates.

An examination of the wavelength dependence of the transient kinetics in this spectral region revels that the red edge of the 530 nm band rises and decays faster than its blue edge: *i.e.*, for the red edge of the band, *τ*(r)tR = 4.2 ps and *τ*(d)tR = 13 ps, corresponding to *k*(r)tR = 2.4 × 10^11^ s^–1^ and *k*(d)tR = 7.7 × 10^10^ s^–1^; and for the blue edge of the band, *τ*(r)tB = 5.3 ps and *τ*(d)tB = 20 ps, corresponding to *k*(r)tB = 1.9 × 10^11^ s^–1^ and *k*(d)tB = 5.0 × 10^10^ s^–1^. (With the signal-to-noise ratio of the kinetic curves, mono-exponential functions can provide statistically acceptable data fits for bi- and multi-exponential decay or rise patterns with time constants that differ with less than about 50%.) An assumption that the blue-edge and the red-edge kinetics of the 530 nm band represent the parallel formation of two different twisted transients from ^1^D*TT, can account for the observed kinetics, *i.e.*, *k*_TT_ ≈ *k*(r)tR + *k*(r)tB; *k*(r)tR ≈ k*nr*i* and *k*(r)tB ≈ k*nr*j*, while *k*_TT_ ≈ k*nr*i* + k*nr*j* (Scheme 3a).

Internal conversion *via* torsional modes of the excited-state twisted conformers, producing ^1^D_TT_, ^1^D_TC_ and ^1^D_CC_, should result in bleach recovery, *k*(r)B, and in growth of the transient absorption band spanning between 440 and 460 nm, *k*(r)C (Table 3). While the rates of decay of the 530 nm transient, *i.e.*, *k*(d)tR and *k*(d)tB corresponding to *k*_ic*i*_ and *k*_ic*j*_ (Scheme 3a), are similar to the rates of bleach recovery, *k*(r1)B = 1/*τ*(r1)B = 6.5 × 10^10^ s^–1^, and of the rise of the 450 nm transient, *k*(r)C = 1/*τ*(r)C = 7.2 × 10^10^ s^–1^, they do not add up. The decay of the potentially two ^1^D*t transients with rate constants ranging between 5 × 10^10^ and 8 × 10^10^ s^–1^ is not fast enough to account for the formation of the three ground-state conformers, each with *k*^(r)^ of about 7 × 10^10^ s^–1^. That is, *k*_ic_ rate constants do not quantitatively add up to the *k*_tr_ ones (Scheme 3a). Therefore, the picosecond non-radiative decay of THIA either leads to the formation of only two of the ground-state planar conformers (^1^D_TT_, ^1^D_TC_ or ^1^D_CC_), or involves additional paths of deactivation to the S_0_ isomers that do not involve the ^1^D*t conformers absorbing at 530 nm.

**Table 3:** Kinetic characteristics of the dynamics of THIA for different alcohol media, obtained from transient-absorption spectroscopy of the S_0_ → S_1_ bleach band of the *trans–trans* conformer, and of the S_0_ → S_1_ absorption band of the two *cis* conformers^*a*^

Solvent^*b*^	*τ* (d) B ^*c*^/ps [*k*(d)B × 10^–10^/s^–1^]	*τ* (r) B ^*d*^/ps (*α*_*i*_) [*k*(r)B × 10^–10^/s^–1^]	*τ* (r) C ^*e*^/ps (*α*_*i*_) [*k*(r)C × 10^–10^/s^–1^]	*τ* (d) C ^*f*^/ps (*α*_*i*_) [*k*(d)C × 10^–10^/s^–1^]
GL	4.1 ± 0.6 [24]^*g*^	250 ± 140 (0.14) [0.40]	75 ± 28 (0.08) [1.3]	>1500 [<0.067]^*h*^
		>1500 (0.86) [<0.067]^*h*^	610 ± 70 (0.91) [0.16]	
EG	32 ± 6 [3.1]	60 ± 9 (0.94) [1.7]	33 ± 4 [3.0]	73 ± 26 (0.92) [1.4]
		>1500 (0.06) [<0.067]^*h*^		>1500 (0.08) [<0.067]^*h*^
MeOH	2.5 ± 0.3 [40]	10 ± 2 (0.84) [10]	10 ± 19 [10]	89 ± 12 (0.52) [1.1]
		76 ± 45 (0.09) [1.3]		770 ± 90 (0.48) [0.13]
		750 ± 110 (0.07) [0.13]		
EtOH	6.0 ± 0.3 [17]	16 ± 1 (0.79) [6.3]	14 ± 2 [7.1]	110 ± 10 (0.45) [0.91]
		110 ± 44 (0.08) [0.91]		780 ± 30 (0.55) [0.13]
		810 ± 120 (0.13) [0.12]		
BuOH	11 ± 1 [9.1]	29 ± 2 (0.89) [3.4]	23 ± 5 [4.3]	210 ± 30 (0.40) [0.48]
		300 ± 80 (0.05) [0.33]		1100 ± 150 (0.60) [0.091]
		1200 ± 170 (0.06) [0.083]		

^*a*^Time constants, *τ*, were extracted from exponential fits. For multiexponential data fits, 
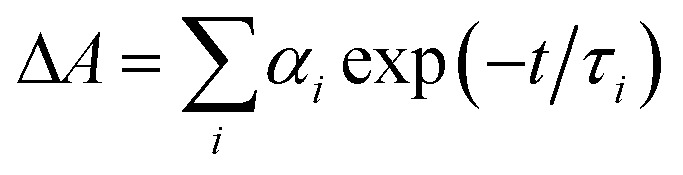
, the relative pre-exponential amplitudes of the same decay or the same rise, *α*_*i*_, are shown in the parentheses. The corresponding rate constants, *k* = 1/*τ*, are shown in the square brackets. The reported values were obtained from exponential data fits of the whole region (*i.e.*, from *t*_0_ = 1 ps to *t* = 3.2 ns). For EG and MeOH, when the data fits were limited to the temporal regions comparable with the expected time constants, the obtained values of *τ* were the same as the ones reported in ref. 47.

^*b*^GL = glycerol; EG = ethylene glycol; MeOH = methanol; EtOH = ethanol; BuOH = 1-butanol.

^*c*^An increase in the magnitude of the bleach band, B. The decay (an increase in the negative Δ*A*) of the bleach band after the initial subpicosecond spike, monitored at the peak value of –Δ*A* between 405 and 445 nm (Fig. 3d).

^*d*^A decrease in the magnitude of the bleach band, B. The picosecond and nanosecond rise components (a decrease in the negative Δ*A*) of the bleach band after the initial subpicosecond spike, monitored at the peak value of –Δ*A* between 405 and 445 nm (Fig. 3d).

^*e*^The formation of the ground-state *cis* conformers, C. The picosecond rise of the kinetic curves recorded at 450 nm.

^*f*^The decay of the ground-state *cis* conformers, C. The picosecond and nanosecond decay of the kinetic curves recorded at 450 nm.

^*g*^For GL, the decay in this spectral region was more pronounced at 450 nm than for the bleach band (Fig. 3c and d).

^*h*^The time constants from the exponential fits exceeded ∼50% of the upper temporal data limit (∼3.2 ns).

The transient-absorption dynamics in the UV region may, indeed, be indicative of an alternative pathway for non-radiative deactivation of the excited state. The analysis suggests for a transient overlapping with the bleach band, which manifests subpicosecond rise and picosecond decay, as indicated by the spike at 430 nm and the consecutive negative increase in Δ*A* with *k*(d)B = 1/*τ*(d)B = 1.7 × 10^11^ s^–1^. This rate of decay is considerably faster than the rates of internal conversion (about 7 × 10^10^ s^–1^), suggesting for the accumulation of yet another transient. The kinetic curve at about 360 nm indicates for spectral overlaps of transients that sequentially decay and rise from one another.^51^ An absorption signature of a third excited-state conformer, concealed in this UV region, allows for “connecting the dots.” That is, a third transient that rises with *k*(d)B ≈ 2 × 10^11^ s^–1^ and decays with *k*_360 nm_ ≈ 7 × 10^10^ s^–1^ (Table 2) plausibly provides a parallel pathway non-radiative deactivation of S_1_ to the ground state. Hence, while *k*(r)t represents two of k*nr, *k*(d)B represents the third k*nr (Scheme 3a). Because the rate constants representing *k*_ic_, *i.e.*, *k*(d)t and *k*_360 nm_ (Scheme 3a), have similar values, we cannot exclude the possibility that the 530 nm band is due to a single ^1^D*t transient and the UV absorption – to two twisted conformers. It should be, also, emphasized that the k*nr, corresponding to *k*(d)B, does not involve relaxation to the fluorescent excited state.

Similar parallel pathways of S_1_ deactivation have been computationally suggested,^52^ implying that the conical intersection, CI (Scheme 2), is not truly “conical.” Instead, CI should represent multiple points in the S_1_ potential-energy plane where internal conversion to S_0_ occurs. Our experimental findings support this mechanistic paradigm, showing three transients consistent with ^1^D_t_ involved in the S_1_ → S_0_ non-radiative deactivation (Scheme 3a).

#### B.4.2. Ground-state dynamics

Because the absorption of the three planar ground-state conformers, ^1^D_TT_, ^1^D_TC_, and ^1^D_CC_ (Scheme 1) considerably overlaps, the measured kinetics of the bleach band represents not only the recovery of ^1^D_TT_ but also the formation and the decay of ^1^D_TC_, and ^1^D_CC_. The ground-state absorption of the two *cis* conformers is shifted to the red of the absorption of ^1^D_TT_. Thus, the band at about 450 nm is conveniently used for monitoring the dynamics of ^1^D_TC_ and ^1^D_CC_.^48^ The absorption of ^1^D_CC_ is shifted even further to the red in comparison with the absorption of ^1^D_TC_.^47^ Hence, we expected a wavelength dependence of the kinetics of the 450 nm band, assigned to the two *cis* conformers.

A kinetic curve monitoring Δ*A* at constant wavelength of 440 nm, exhibits a rise with *τ*(r)440 nm = 11 ± 1 ps and a biexponential decay with *τ*(d1)440 nm = 120 ± 10 ps (*α*_1_ = 0.38), *τ*(d2)440 nm = 820 ± 35 ps (*α*_2_ = 0.62), corresponding to *k*(r)440 nm = 9.1 × 10^10^ s^–1^, *k*(d1)440 nm = 8.3 × 10^9^ s^–1^, and *k*(d2)440 nm = 1.2 × 10^9^ s^–1^ (Table 3). This kinetic behaviour is consistent with the rise and the decay of the two ground-state *cis* conformers. Concurrently, the data fits of the kinetic curves recorded at red edge of the same band yield only the slow decay components: *e.g.*, *τ*(d)470 nm = 750 ± 20 ps, *τ*(d)480 nm = 780 ± 40 ps, and *τ*(d)490 nm = 880 ± 140 ps. Therefore, the red edge of the band corresponds to the ground-state absorption of the *cis–cis* conformer. This finding confirms that the slow kinetic components of *k*(d)C and *k*(r)B (∼10^9^ s^–1^) represent the transition of ^1^D_CC_ to ^1^D_TT_, (*i.e.*, *k*_nr2_ or *k*_nr3_, Scheme 3b), and the fast components (∼10^10^ s^–1^) correspond to the conversion of ^1^D_TC_ to ^1^D_TT_ (*i.e.*, *k*_nr1_, Scheme 3b).

The conversion of ^1^D_TC_ to ^1^D_TT_ requires a single-step rotation of one of the rings around the partially π-conjugated linker. The transition of ^1^D_CC_ to ^1^D_TT_, however, can involve either a stepwise, ^1^D_CC_ → ^1^D_TC_ → ^1^D_TT_, or concerted single-step, ^1^D_CC_ → ^1^D_TT_, pathway (Scheme 3b). Because ^1^D_TC_ → ^1^D_TT_ is faster than ^1^D_CC_ → ^1^D_TT_, it cannot be discerned if the measured slow components of *k*(d)C and *k*(r)B represent the rate-limiting step, ^1^D_CC_ → ^1^D_TC_ (*k*_nr2_), of a stepwise mechanism, or the rate of a concerted transformation, ^1^D_CC_ → ^1^D_TT_ (*k*_nr3_) (Scheme 3b). Indeed, a concerted pathway involving simultaneous rotation of both rings proceeds through a twisted *staggered–staggered syn* conformer with pronouncedly localized positive charge on the methine carbon of the linker (Scheme 1). Such polarized conformers are electrostatically less favourable than the singly *staggered* twisted rotamers, involved in the stepwise transformations, with only partial localization of the positive charge on one of the nitrogens (Scheme 1). Nevertheless, this electrostatic argument cannot completely rule out possible concerted pathways for transforming the *cis–cis* into *trans–trans* conformers, considering a relatively polar liquid media that can reorganize within the timescales of the molecular ring rotations of THIA and solvate the transient localized charges.

### B.5. Solvent effects on the transient dynamics

An increase in solvent viscosity increases the emission lifetime of THIA by decreasing the rates of non-radiative decay (Fig. 1c, Tables 1 and 2). This solvent dependence, however, differs for the various processes involved in the S_1_ deactivation and viscosity by itself does not account for all observed trends.

The solvent media affects the accumulation of the 530 nm transient, ascribed to the twisted conformers, ^1^D*t, involved in the internal conversion of S_1_ to S_0_. Changing the solvent from methanol (MeOH) to ethanol (EtOH) and to 1-butanol (BuOH) delays the build-up of these transients and causes a decrease in the maximum Δ*A* observed at 530 nm (Fig. 3b).

For the two most viscous solvents, ethylene glycol and glycerol, we did not observe a transient at 530 nm (Fig. 3a and b), which suggests that either (1) the deactivation of the excited to the ground state does not involve the 530 nm transient, or (2) the formation of ^1^D*t becomes the rate limiting step, *i.e.*, k*nr < *k*_ic_ (Scheme 3a), and does not allow for accumulation of the twisted excited-state transient that absorb at 530 nm. The kinetic analysis shows that the latter is the case for ethylene glycol and glycerol.

The formation of twisted conformers involves rotation of the aromatic rings around the methine linker. Media viscosity impedes such molecular motions. Conversely, the decay of ^1^D*t involves internal conversion, IC, forming high-energy ground-state twisted rotamers, ^1^D_t_, that relax to one of the three planar conformers (Scheme 3a). IC does not involve large structural changes, and hence, it should not be strongly affected by the media viscosity. On the contrary to this expectation, the decay rates of ^1^D*t appears to decrease with an increase of solvent viscosity (*k*(d)t, Table 2). The interpretation of these kinetic trends for MeOH, EtOH and BuOH, however, ought to be approach with caution because the increase in viscosity accompanies a decrease in media polarity for this solvent series. In addition to viscosity effects, therefore, the experimental finding can also indicate that the rates of formation and decay of ^1^D*t decrease with a decrease in solvent polarity. As an alternative, water is more viscous than MeOH and less viscous than EtOH. Also, water is more polar than any of the alcohols. For THIA in water we observed the formation of the 530 nm transient with *k*(r)t = 3.6 × 10^11^ s^–1^, which is smaller than *k*(r)t for MeOH and larger than *k*(r)t for EtOH (Table 2), *i.e.*, it agrees well with the expected trend for solvent viscosity. The decay rate constant of the 530 nm transient, *k*(d)t, for water, however, is 1.7 × 10^11^ s^–1^, which is larger even than that for MeOH. Hence, the media polarity, rather than viscosity, dominates the internal conversion involved in the decay of ^1^D*t.

To further prove that the viscosity and the polarity of the solvent media govern the photophysics of THIA, we employed acetonitrile (MeCN) for some of the studies. MeCN is an aprotic solvent that is less viscous than MeOH. For MeCN, the photoexcited dye exhibits faster kinetics of deactivation than for MeOH, which follows the viscosity-dependence trends for the five alcohols (Fig. 3b–d). Therefore, we unequivocally can eliminate the possibility for detectable effects of the protic nature of the alcohol media on the photophysics of THIA.

The structural features of the cyanine dye provide an explanation for this polarity dependence of the IC rates. While the molecular structure of THIA is symmetric, in the twisted conformers the π-conjugation at the linker is interrupted and the positive charge partially localizes on one of the nitrogen heteroatoms or on the methine carbon (Scheme 1).^50^ Such a decrease in the effective radius of the charge distribution^67^ increases the solvation energy of the dye.^68^–^70^ Hence, the solvation alters the potential-energy profiles at the interfaces between S_1_ and S_0_, changing in the rates of internal conversion.

Projecting this polarity trend to glycerol and ethylene glycol, which are more polar than the other three alcohols (Table 1), one would expect an increase in the decay rates for the 530 nm transient. Concurrently, the increased viscosity of glycerol and ethylene glycol impedes the molecular ring rotation, making the formation of the 530 nm transient the rate-limiting step of this route of decay of S_1_ to S_0_. Thus, the simultaneous decrease of *k*(r)t and increase of *k*(d)t prevent the accumulation of ^1^D*t (absorbing at 530 nm), which is consistent with the observed transient spectral features for glycerol and ethylene glycol (Fig. 3a and b).

While we did not observe 530 nm transient for glycerol and ethylene glycol, THIA still exhibits transient absorption in the 340–360 nm even for these two viscous solvents (Fig. 3a). It confirms that these UV transients most plausibly represent an alternative pathway of S_1_ deactivation that is independent from the 530 nm transients. Indeed, the rates of internal conversion through this alternative pathway are slower than the rates of the formation of the transient absorbing around 360 nm.

**Scheme 4:**
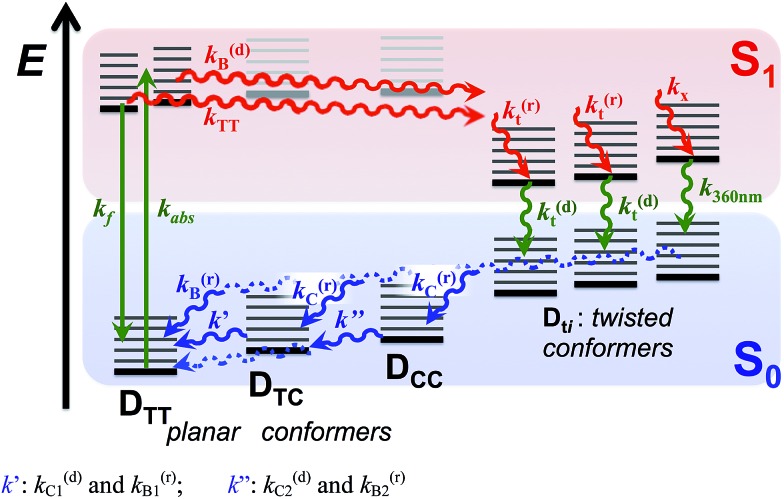
Excited- and ground-state dynamics of THIA as determined from transient-absorption kinetics. *k*′: *k*(d)C1 and *k*(r)B1; *k*′′: *k*(d)C2 and *k*(r)B2.

A mechanistic scheme with parallel pathways for the formation of three twisted transients requires *k*_TT_ + *k*(d)B ≈ 2*k*(r)t + *k*_*x*_, where *k*_TT_ represents the decay of the emissive conformer, ^1^D*TT, and *k*(d)B – the decay of the excited-state transient that has UV absorption overlapping with the ground-state absorption of the *trans–trans* THIA. 2*k*(r)t accounts for the formation of the two twisted transients that absorb at 530 nm, and *k*_*x*_ represents the rise of the transient that decays with *k*_360 nm_ (Tables 2 and 3, and Schemes 3 and 4). For MeOH, EtOH and BuOH, *k*_*x*_ has to assume values of about 2.7 × 10^11^ s^–1^, 3.2 × 10^11^ s^–1^, and 7.1 × 10^10^ s^–1^, respectively, according to such a mechanism.

Concurrently, if the three excited-state twisted conformers decay to all three ground-state planar rotamers, *k*_360 nm_ + 2*k*(d)t ≈ 2*k*(r)C + *k*(r1)B. That is, the decays *k*_360 nm_ of the UV-absorbing transient and the decays *k*(d)t of the two transients absorbing at 530 nm should produce the two ground-state planar *cis* rotamers with *k*(r)C each, and the *trans–trans* conformer leading to the bleach recovery with *k*(r1)B. Within the experimental errors, the above equality condition required for this kinetic scheme is valid for MeOH, EtOH and BuOH (Tables 2 and 3).

For ethylene glycol and glycerol, the 530 nm transients do not accumulate, and hence, *k*(r)t and *k*(d)t cannot be determined. To elucidate the pathways that involve the twisted conformers, therefore, we resort to information about the processes yielding the three ^1^D*t and about the transients to which the decays of ^1^D*t leads. For the former, k*nr1 + k*nr2 + k*nr3 = *k*_TT_ + *k*(d)B – *k*_f_ (Scheme 3b) represents the total rate of formation of the three twisted conformers. Cumulatively, the decays of the ^1^D*t transients, *k*_ic1_ + *k*_ic2_ + *k*_ic3_, lead, *via* non-radiative pathways, to the three ground-state planar conformers, 2*k*(r)C + *k*(r1)B – *k*_f_. Therefore, when 2*k*(r)C + *k*(r1)B is much smaller than *k*_TT_ + *k*(d)B, the twisted conformers accumulate as observed for MeOH, EtOH and BuOH. In the case of ethylene glycol and glycerol, the ^1^D*t transients absorbing at 530 nm are not observed, but the one absorbing around 360 nm is still apparent. Therefore, even for these two viscous solvents, 2*k*(r)C + *k*(r1)B has to be smaller than *k*_TT_ + *k*(d)B.

For ethylene glycol, 2*k*(r)C + *k*(r1)B, amounts to 7.7 × 10^10^ s^–1^, and *k*_TT_ + *k*(d)B to 6.4 × 10^10^ s^–1^. That is, 2*k*(r)C + *k*(r1)B is not smaller than *k*_TT_ + *k*(d)B. If 2*k*(r)C + *k*(r1)B ≈ *k*_TT_ + *k*(d)B, the formation of the twisted conformers is the rate-limiting step and no ^1^D*t transient can be observed. The 360 nm transient, however, is apparent for EG media. This discrepancy for EG, *i.e.*, 2*k*(r)C + *k*(r1)B > *k*_TT_ + *k*(d)B, requires an alternative mechanistic consideration. For example, an assumption that the non-radiative decay of S_1_ leads to only two ground-state planar conformers can address this discrepancy, as accommodated by *k*(r)C + *k*(r1)B < *k*_TT_ + *k*(d)B, or 2*k*(r)C < *k*_TT_ + *k*(d)B.

The bleach recovery of THIA in EG exhibits a biexponential character. The two rate constants, *k*(r1)B and *k*(r2)B, match agreeably the decay constants of the *cis* transients, *k*(d1)C and *k*(d2)C (Table 3). This result suggests that the non-radiative deactivation of S_1_ in EG does not lead to the formation of the ground-state *trans–trans* conformer, ^1^D_TT_. Conversely, the bleach recovery would be biexponential (instead of triexponential as observed for the three non-viscous alcohols) if coincidentally the rate of formation of ^1^D_TT_ from S_1_ were similar to the rate of transformation of ^1^D_TC_ (or ^1^D_CC_) to ^1^D_TT_. Furthermore, the slow components of the 450 nm decay, *k*(d2)C, and of the bleach recovery, *k*(r2)B, represent a small contribution to the biexponential kinetics (Table 3). Nevertheless, the wavelength dependence of the decay kinetics reveals an increase in its relative contribution of the slow component at the very red edge of the 450 nm transient band, which is consistent with ascribing the nanosecond *k*(d)C and *k*(r)B to the transition from ^1^D_CC_ to ^1^D_TT_ (Scheme 3b). The picosecond process, characterized by the fast components of *k*(d)C and *k*(r)B for ethylene glycol, is thus ascribed to the ^1^D_TC_ → ^1^D_TT_ transition. For the ethylene glycol media, therefore, the torsional relaxation of the excited state to ^1^D_TT_ is undetectable.

For glycerol, the decays of the emissive ^1^D*TT transient and the rise of the ground-state planar *cis* transients manifest heterogeneous kinetics, as evident from the biexponential fits (Tables 2 and 3). The slight decay in the violet region of the spectrum, *k*(d)B, is quite fast and does not match any of the other rise or decay kinetics for glycerol (Tables 2 and 3). Contributing to the formation of the 360 nm long-lived conformer presents a plausible fate of the fast decaying transient characterized with *k*(d)B. This fast feature, *k*(d)B, along with the heterogeneity of the decay kinetics, indicates that the glycerol media allows pathways of deactivation, which are not viscosity dependent.

To match the rate constants for glycerol, it is feasible to assume that the S_1_ deactivation leads to the formation of ^1^D_TT_ and only one of the ground-state *cis* planar conformers. That is, for the fast (minor) components, *k*_TT,1_ is approximately equal to *k*(r1)B + *k*(r1)C, and not to *k*(r1)B + 2*k*(r1)C or to 2*k*(r1)C. Similarly, for the slow (major) components, *k*_TT,2_ + *k*_360 nm_ ≈ *k*_f_ + *k*(r2)C, where the radiative decay, *k*_f_ (Table 1) and the decay of the *cis* transient, *k*(d)C, contribute to the slow bleach recovery (Table 3).

Because the timescales of the pump-probe measurements are limited to about 3 ns, for glycerol and ethylene glycol, it is challenging to quantify nanosecond time constants (*e.g.*, *k* < 6.7 × 10^–8^ s^–1^, Table 3). Although a monoexponential function describes the slow decay for glycerol, one cannot rule out a possibility of multiexponential character with two or more nanosecond time constants averaged as *τ*(d)C. Therefore, it is quite plausible that the decay of the ground-state *cis* transient(s), *τ*(d)C, is biexponential, and the bleach recovery, *τ*(r)B, is triexponential with two nanosecond components.

Regardless of the number of nanosecond components, the ground-state transformation of the *cis* conformers to the *trans–trans* isomer represents the principal contribution to the bleach recovery for glycerol. For ethylene glycol, the bleach recovery is also dominated by the transformation of ^1^D_CC_ and ^1^D_TC_ to ^1^D_TT_. These trends suggest that suppressing the direct relaxation of the S_1_ state to the *trans–trans* ground state increases the fluorescence quantum yield of THIA.

The ground-sate relaxation of the planar *cis* rotamers to the stable *trans–trans* conformer exhibits dependence not only on the viscosity but also on the polarity of the media. The decay rate constants of the *cis* conformers, *k*(d)C match the recovery of the *trans–trans* absorption, *k*(r)B (Table 3). The rate constants corresponding to the slow components of this transformation, *i.e.*, of the transition of ^1^D_CC_ to ^1^D_TT_, are almost the same, ∼1 × 10^9^ s^–1^, for the three less viscous solvents. Glycerol and ethylene glycol cause a drop in the rate constants below ∼7 × 10^8^ s^–1^, which is consistent with viscosity impeded *cis* to *trans* torsional conversions.

The rates of the fast components corresponding to the transition of ^1^D_TC_ to ^1^D_TT_, however, do not manifest the same viscosity-dependence trend. Changing the solvent from MeOH, to EtOH and to BuOH decreases the fast-component rates (Table 3). This transition from MeOH to BuOH corresponds to a simultaneous increase in viscosity and a decrease in polarity (Table 1). For ethylene glycol, which is more polar than MeOH, however, the rate corresponding to the fast component of the ground-state transition is faster than that for MeOH (Table 3), indicating that media polarity has dominant effect on the transition from the *trans–cis* to the *trans–trans* conformer.

For glycerol, we do not observe a sub-nanosecond component for the transition from the ground-state *cis* rotamers to the *trans–trans* conformer. A slow non-radiative decay of the excited state, *e.g.*, *k*_360 nm_ for glycerol (Table 2), can make the S_1_ → S_0_ transition the rate-limiting step of ^1^D* → ^1^D_TC/CC_ → ^1^D_TT_, preventing a build-up of planar *cis* S_0_ conformers.

These trends indicate that while predominantly the solvent viscosity governs the relaxation of the ground-state *cis–cis* conformer, the media polarity has a measureable effect on the conversion of the *trans–cis* to the *trans–trans* conformer.

## C. Discussion

The solvent dependence of the transient-absorption kinetics of THIA reveals the complexity of its photophysics that can be explained by multiple parallel pathways for transition from the excited to the ground state (Schemes 3 and 4). The media viscosity and polarity not only affect the rates of the various steps of deactivation of the photoexcited dye, but also alter the sequence of processes that dominated the photophysics of THIA.

While viscous media favours the torsional transitions that involve minimal changes in the shape of the solvation cavity of THIA, the non-polar media favours the twisted structures with minimal charge localization. Therefore, the conversion of the planar ^1^D*TT to the *staggered–staggered syn*^1^D*t should dominate the kinetics of THIA in viscous solvents.

In non-polar media, ^1^D*TT should preferably deactivate *via trans-staggered*^1^D*t, if solvation exerts dominating effects. Indeed, such transitions may involve only partially twisted rotamers that provide proximity between S_1_ and S_0_ just enough to attain efficient internal conversion, but do not cause significant charge localization to destabilize the twisted structures. Once in the S_0_ state, such partially twisted structures produced from ^1^D*TT, should plausibly relax predominantly to ^1^D_TT_. Indeed, the structures on Scheme 1 represent only the “extreme examples” of planar and 90°-twisted conformers, and the transients observed at 530 and 360 nm are most likely “intermediate” partially twisted rotamers.

For MeOH, EtOH and BuOH, the fastest component of the bleach recovery, *k*(r1)B, dominates the kinetics of relaxation from the excited state (Table 3). For THIA in water and acetonitrile, the fastest component, *k*(r1)B, also dominates the bleach recovery. That is, regardless of polarity, non-viscous media favours the deactivation of S_1_ leading directly from ^1^D*t to the most stable planar structure, ^1^D_TT_. For the non-viscous non-polar alcohols, preferential transitions *via* conformers with minimum charge localization can account for the observed relaxation of S_1_ to ^1^D_TT_.

For glycerol, the formation of the *cis* planar conformers dominates the relaxation to S_0_ as evident from the major contribution from the slow component, *k*(r2)B (Table 3). Because glycerol is the most viscous and most polar of the five alcohols, this trend suggests that the relaxation of S_1_ to the ground-state planar *cis* conformers involves minimum change in the shape of the solvation cavity, regardless of charge localization during the torsional motions. Indeed, synchronized rotation of multiple bonds allows *cis–trans* transitions of polymethine structures in the confinement of protein media.^71^,^72^ This rationale suggests that for viscous media the *trans–trans* excited state, ^1^D*TT, preferentially relaxes to the *staggered–staggered syn*^1^D*t conformer without significant perturbation of the shape of the solvation cavity. Following the same direction of ring rotation needed for the disrotatory transition from the *trans–trans* to the *staggered–staggered syn* structure (Scheme 1) yields a *cis–cis* conformer. This scheme involving minimal solvation distortion, *i.e.*, ^1^D*TT → ^1^D*t(*syn*) → ^1^D_t_(*syn*) → ^1^D_CC_, agrees with the kinetic data for glycerol and ethylene glycol should the 530 nm absorption be due to the *staggered–staggered* conformers that do not accumulate when the internal conversion is not the rate-limiting step, *i.e.*, k*nr < *k*_ic_ (Scheme 3a).

The solvent viscosity has a dominating effect on the emission properties of THIA and correlates well with its florescence quantum yield (Table 1, Fig. 1). The effects of the media polarity, however, should not be overlooked. Viscous media suppresses the different torsional modes responsible for the depletion of the fluorescent ^1^D*TT conformer from its local energy minimum on the S_1_ potential-energy plane. It is consistent with the increase in its lifetime, *τ*_TT_, as the solvent viscosity increases (Table 1). Conversely, the media polarity is essential for stabilizing the *staggered–staggered* twisted conformers that provide the only possible pathways to ^1^D_CC_ (Schemes 1 and 3). The two relatively polar and viscous alcohols cause an orders-of-magnitude increase in *Φ*_f_ of THIA. For these two alcohols, the pathways leading to the ground-state *trans–cis* and *cis–cis* isomers dominate the decay of the excited state.

These findings have a key implication for molecular design of cyanine fluorescent photoprobes. Covalent locking of the *trans–trans* structures is a feasible approach for suppressing the formation of the twisted conformers that is responsible for non-radiative deactivation. As a simple alternative, introducing long chains, instead of ethyls, to the aromatic rings may offer a means for suppressing the generation of the twisted conformers leading to direct relaxation to ^1^D_TT_, while forming the *staggered–staggered syn*^1^D*t, yielding ^1^D_CC_, would not strongly affect *Φ*_f_.

Overall, the multiple parallel decay pathways govern the photophysics of THIA. The observed trends provide an insight in the polarity and viscosity dependence of the different routes of deactivation of photoexcited polymethine conjugates. For monomethine dyes, such as THIA, the trends reveal that non-radiative pathways that lead to the decay of the S_1_ state directly to the *trans–trans* ground-state planar conformer, ^1^D_TT_, provide the principal competition to the radiative decay. That is, such a non-radiative pathway is responsible for quenching the fluorescence and for decreasing the emission quantum yields.

## D. Conclusions

The numerous torsional modes involving rotations around the methine bonds for even the simplest cyanine dyes result in a pronounced complexity in their photophysics. Indeed, generalized simplistic mechanistic schemes (Scheme 2) cannot capture the genuine complexity of the multiple parallel pathways of deactivation of the electronically excited state. Even for one of the simplest cyanine dyes, THIA, the underlying complexity of its photophysics carries a wealth of mechanistic information (Schemes 3 and 4), which can prove important in the design of photoprobes for biological, medical and materials applications.

## E. Experimental

### E.1. Materials

Coumarin 151 (C151) and 3,3′′-diethylthiacyanine (THIA) iodide was purchased from Sigma-Aldrich. Spectroscopic grade solvents, methanol, ethanol, 1-butanol, ethylene glycol and glycerol, were obtained from Fisher Scientific. Each sample was prepared from a fresh stock solution of THIA or C151 in ethanol, by diluting in the pertinent solvent. The dilutions of the non-ethanol samples were carried out in a manner ensuring that the final concentration of ethanol from the stock solution does not exceed ∼0.5%. Except for the two viscous solvents, ethylene glycol and glycerol, the samples were purged with argon (∼10 min per ml of sample) in sealed quartz cuvettes prior to each measurement.

### E.2. Methods

Steady-state absorption spectra were recorded in a transmission mode using a JASCO V-670 spectrophotometer (Tokyo, Japan); and steady-state emission spectra were measured, also in a transmission mode, with a FluoroLog-3 spectrofluorometer (Horiba-Jobin-Yvon, Edison, NJ, USA) as previously reported.^73^,^74^ The fluorescence quantum yields, *Φ*_f_, of THIA for the different solvents were calculated from the emission and absorption spectral data,^75^ using samples of C151 dissolved in ethanol for a standard (*Φ*_0_ = 0.49).^76^ The transient-absorption measurements were recorded in transmission mode using a Helios pump-probe spectrometer (Ultrafast Systems, LLC, Florida, USA).^77^,^78^ The laser source for the Helios was a SpitFire Pro 35F regenerative amplifier yielding (Spectra Physics, Newport, CA, USA) generating 800 nm pulses (>35 fs, 4.0 mJ, at 1 kHz). The amplifier was pumped with an Empower 30 Q-switched laser ran at 20 W. A MaiTai SP oscillator provided the seed beam (55 nm bandwidth).^79^ The wavelength of the pump was tuned using an optical parametric amplifier, OPA-800CU (Newport Corporation, Newport, CA, USA), equipped with a signal second and forth harmonic generators.^77^,^78^ Responses from pure solvents were used for the chirp correction of the transient-absorption data, Δ*A*(*t*, *λ*). The data analysis was carried out using IgorPro v. 6 (WaveMetrics, Inc., Lake Oswego, OR, USA).^63^,^80^

### E.3. Analysis of transient-absorption kinetics and rationale

The pronounced wavelength shifts of the bands during the evolution of the transient-absorption spectra (Fig. 2) are indicative of the conformational changes of the cyanine molecules as they “migrated” along the energy planes of the ground and excited states. As a result of these spectral shifts, and of the pronounced overlaps between the different transient-absorption bands, the kinetic curves at most wavelengths of interest exhibit multiexponential patterns yielding a wide range of rate constants that could not be always directly related to one another. This complexity of the transient absorption dynamics of THIA prevented a reliable use of global-fitting algorithms. For each kinetic curve, therefore, we used a minimum number of exponential terms that provided a good data fit. Furthermore, for the initial estimate of the kinetic features of the photoinduced dynamics of THIA, we also monitored the evolution of each spectral band at its maximum or minimum, in addition to recording Δ*A*(*t*) at a constant wavelength.

#### E.3.1. Subpicosecond dynamics

Upon photoexcitation, fast rises and decays of the UV transient-absorption band monitored at 360 nm and of the bleach band at about 430 nm resemble coherence spikes (Fig. 2c). Such spikes near the time zero can be ascribed to: (1) coherent-coupling “artifacts,”^81^–^84^ reported for other cyanine dyes,^85^,^86^ representing the properties of the pump and the probe pulses, rather than the photoinduced dynamics of the analyzed chromophores; (2) non-resonance coherent response from the solvent, such as the electronic response preceding the relatively weak signal from the impulsive stimulated Raman scattering;^87^ and (3) resonance coherent responses from the solute, THIA. The excitation pulses we use are depolarized and have relatively broad bandwidths (∼600 cm^–1^). The magnitude of the observed spikes for the THIA samples is larger than the magnitude of the features from the measurements for the chirp correction that used neat solvents. Also, the spikes at time zero appear only in the spectral region of the ground-state absorption of THIA, implying a resonance origin of these features. Thus, we can exclude the first two options as a possibility for the origin of the femtosecond spikes.

The femtosecond solute response at wavelengths shorter than about 430 nm can be ascribed to: (1) redistribution of the ground-state population, recovering conformers responsible for the red-edge ground-state absorption; (2) evolution of the excited-state population, including a rise of a transient that also exhibits absorption around 430 nm that overlaps with the S_1_ ← S_0_ spectral band of the dye; (3) subpicosecond internal conversion providing transition from the excited to the ground *trans–trans* state; and (4) resonance coherent processes involving THIA, which ought to be most pronounced for times shorter than about 100 fs, *i.e.*, at optimal pump-probe overlap. Potential contributions from coherent signals pose challenges to quantifying transient absorption kinetics with time constants smaller than about 100 or 200 fs.

The reshaping of the of the bleach band within the first one picosecond after the excitation (Fig. 2a and c) can result from redistribution of the ground state population and/or from an overlap of the ground-state absorption, S_1_ ← S_0_, with the absorption of excited-state transients, S_1+*n*_ ← S_1_, which manifests a subpicosecond rise. The stimulated emission and the 730 nm transient grew simultaneously to reach maximum at about 600–700 fs after the excitation. A blue shift in the stimulated emission accompanies the band growth and proceeds beyond 1 ps after the excitation (Fig. 2a). This evolution of the spectral bands is consistent with subpicosecond relaxation of the nuclear wave packet in the excited state to the local minimum corresponding to the fluorescent *trans–trans* conformer, ^1^D*TT (Scheme 2).

#### E.3.2. Picosecond dynamics

The broad transient absorption band at 730 nm decays monoexponentially, *τ*_TT_ = 2.2 ± 0.1 ps (Table 2). Concurrently, the evolution of the stimulated emission, monitored at the band minimum, exhibits a biexponential rise, *τ*(r1)SE = 2.2 ± 0.2 ps (*α*_1_ = 0.38), *τ*(r2)SE = 5.2 ± 0.5 ps (*α*_2_ = 0.62), leading to Δ*A* values larger than zero, which decay monoexponentially, *τ*(d)SE = 14.8 ± 0.7 ps (Table 2). Positive Δ*A* in the fluorescence spectral region is an indication for the growth and the decay of a transient with absorption overlapping with the emission of THIA. The equality between *τ*_TT_ and one of the stimulated-emission rise components, *τ*(r1)SE, confirms the assignment of the 730 nm band to the *trans–trans* singlet excited state, ^1^D*TT, responsible for the radiative deactivation of the S_1_ state. Therefore, the decay time constant of the 730 nm broad transient-absorption band, *i.e.*, *τ*_TT_ (Tables 1 and 2), represents the fluorescence lifetime of THIA. The decay, *τ*(d)SE, and the other rise component, *τ*(r2)SE, represent the dynamics of one or more transients absorbing in the spectral region of the emission band (Table 2).

A data fit of the kinetic curve monitoring the maximum of the 530 nm band, yields a rise *τ*(r)t = 5.0 ± 0.2 ps, and a decay *τ*(d)t = 13 ± 1 ps (Table 2). These two time constants match *τ*(r2)SE and *τ*(d)SE from the fit of the kinetics recorded at stimulated-emission region, suggesting that they most likely correspond to the rise and the decay of the same transient(s) with absorption extending between about 470 and 580 nm.

The kinetic curve following the maximum of the band between 440 and 460 nm exhibits somewhat complex dynamics. Two features, however, stand out in the span between 10 ps and 3 ns after the excitation (Fig. 2b and c): a pronounced rise with *τ*(r)C = 13.8 ± 1.8 ps followed by biexponential decay with *τ*(d1)C = 111 ± 8 ps (*α*_1_ = 0.45), *τ*(d2)C = 780 ± 30 ps (*α*_2_ = 0.55). (Monoexponential analysis did not yield reasonable fits for the kinetics of the 450 nm band.)

The decay constant of the 530 nm transients, *τ*(d)t, matches the rise constant, *τ*(r)C, of the absorption at ∼450 nm assigned to the S_1_ ← S_0_ transition of the two ground-state *cis* conformers (Table 2). It confirms that the 530 nm transient-absorption band corresponds to one or more conformers, ^1^D*t, through which the *trans–trans* S_1_ state decays to the *cis* S_0_ states. Hence, *τ*(d)t and *τ*(r)C characterize the rates of internal conversion, *k*_ic_, at the interfaces between the S_1_ and S_0_ states (Scheme 3a).

After the subpicosecond spike, the bleach band in the 390–440 nm spectral region exhibits more than 50% negative growth with *τ*(d)B = 6.0 ± 0.3 ps (Fig. 2c, Table 2). This increase in |–Δ*A*| may suggest for picosecond depletion of the *trans–trans* ground state of THIA. The *trans–trans* conformer, however, is at the minimum of the S_0_ potential-energy plane, and no intramolecular spontaneous process could possibly deplete it picoseconds after the photoexcitation. As an intermolecular alternative, to account for the picosecond depletion of the absorption of the *trans–trans* ground state at 30 μM concentration, for example, excimer formation requires rates with *k* ≈ 6 × 10^15^ M^–1^ s^–1^, which is absurdly impossible for any diffusion-limited process in condensed phase. Therefore, this 6 ps negative increase in Δ*A* cannot result from depletion of the ground-state absorption of the *trans–trans* conformer. Instead, it can plausibly originate from the decay of a transient that absorbs in the spectral region of the bleach band.

#### E.3.3. Subnanosecond dynamics

At about 7 ps after the excitation, the bleach band grows to its maximum magnitude of |–Δ*A*|. The consequent decrease in |–Δ*A*| to baseline manifests triexponential kinetics with *τ*(r1)B = 15.5 ± 0.9 ps (*α*_1_ = 0.79), *τ*(r2)B = 113 ± 44 ps (*α*_2_ = 0.08), and *τ*(r3)B = 810 ± 120 ps (*α*_3_ = 0.13). The two minor components, *τ*(r2)B and *τ*(r3)B, match the decay components of the 450 nm band (Table 2), hence, we ascribe them to the recovery of the *trans–trans* ground-state *via* torsional transitions of the two ground-state *cis* isomers. Conversely, the major component, *τ*(r1)B, can represent the kinetics of: (1) non-radiative recovery of the *trans–trans* ground-state, ^1^D_TT_, directly from the excited state; and/or (2) a growth of the S_1_ ← S_0_ absorption of the two *cis* conformers that overlaps with the *trans–trans* ground-state absorption.

Although THIA is one of the simplest cyanine dyes, the deactivation of its excited states is quite complex. For a holistic mechanistic view, it is crucial to quantify the relationships between the rise and decay kinetics of the observed transients.
