# Correction: Photoinduced dynamics of a cyanine dye: parallel pathways of non-radiative deactivation involving multiple excited-state twisted transients

**DOI:** 10.1039/c5sc90020d

**Published:** 2015-04-09

**Authors:** Srigokul Upadhyayula, Vicente Nuñez, Eli M. Espinoza, Jillian M. Larsen, Duoduo Bao, Dewen Shi, Jenny T. Mac, Bahman Anvari, Valentine I. Vullev

**Affiliations:** a Department of Bioengineering , University of California , Riverside , CA 92521 , USA . Email: vullev@ucr.edu; b Department of Biochemistry , University of California , Riverside , CA 92521 , USA; c Department of Chemistry , University of California , Riverside , CA 92521 , USA; d Materials Science and Engineering Program , University of California , Riverside , CA 92521 , USA

## Abstract

Correction for ‘Photoinduced dynamics of a cyanine dye: parallel pathways of non-radiative deactivation involving multiple excited-state twisted transients’ by Srigokul Upadhyayula *et al.*, *Chem. Sci.*, 2015, **6**, 2237–2251.



## 

Fig. 3d was displayed incorrectly; the correct version of Fig. 3 appears below.
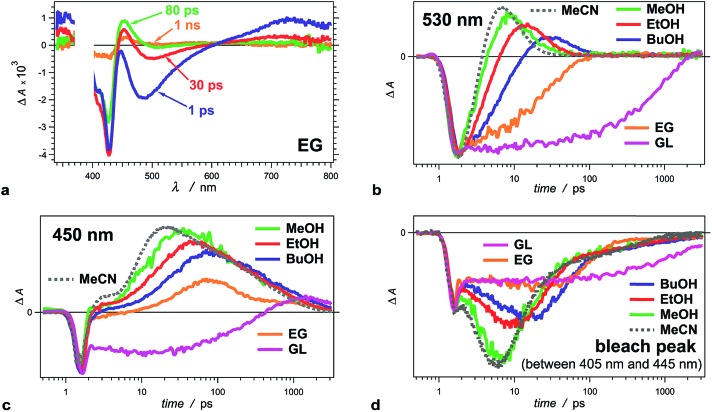


The Royal Society of Chemistry apologises for these errors and any consequent inconvenience to authors and readers.

